# Preparation of Pd/Bacterial Cellulose Hybrid Nanofibers for Dopamine Detection

**DOI:** 10.3390/molecules21050618

**Published:** 2016-05-11

**Authors:** Dawei Li, Kelong Ao, Qingqing Wang, Pengfei Lv, Qufu Wei

**Affiliations:** Key Laboratory of Eco–Textiles, Ministry of Education, Jiangnan University, Wuxi 214122, Jiang Su, China; ldw19900323@163.com (D.L.); 1090112401@vip.jiangnan.edu.cn (K.A.); wqq888217@126.com (Q.W.); 6130703014@vip.jiangnan.edu.cn (P.L.)

**Keywords:** palladium nanoparticle, bacterial cellulose, laccase, dopamine, biosensor

## Abstract

Palladium nanoparticle-bacterial cellulose (PdBC) hybrid nanofibers were synthesized by *in-situ* chemical reduction method. The obtained PdBC nanofibers were characterized by a series of analytical techniques. The results revealed that Pd nanoparticles were evenly dispersed on the surfaces of BC nanofibers. Then, the as-prepared PdBC nanofibers were mixed with laccase (Lac) and Nafion to obtain mixture suspension, which was further modified on electrode surface to construct novel biosensing platform. Finally, the prepared electrochemical biosensor was employed to detect dopamine. The analysis result was satisfactory, the sensor showed excellent electrocatalysis towards dopamine with high sensitivity (38.4 µA·mM^−1^), low detection limit (1.26 µM), and wide linear range (5–167 µM). Moreover, the biosensor also showed good repeatability, reproducibility, selectivity and stability and was successfully used in the detection of dopamine in human urine, thus providing a promising method for dopamine analysis in clinical application.

## 1. Introduction

Dopamine (DA) is an important neurotransmitter, which plays pivotal roles in the central nervous, cardiovascular and hormonal systems [1]. The abnormity of dopaminergic neurotransmission can lead to some serious consequences, like attention deficit, schizophrenia, mood disorders, hyperactivity disorder, and neurodegenerative diseases such as Parkinson’s disease and Alzheimer’s disease [2]. Hence, it is of importance to develop highly efficient, rapid and precise analytical methods to monitor the concentration of DA in human bodies. Up to now, a variety of analytical techniques have been invented for the determination of DA, including optical spectroscopy [3], mass spectrometry [4], high performance liquid chromatography (HPLC) [5], and electrochemical methods [6,7]. Among these methods, electrochemical biosensors have attracted wide attention due to the great advantages of enzyme biosensors over conventional analytical techniques, like low price, high sensitivity/selectivity, rapid response and amenable miniaturization. Laccase (Lac), as a multicopper oxidase, can catalyze the DA to its oxidation form accompanied by reduction of molecular. Based on this, a large number of Lac-based biosensors for the detection of DA have been reported [8,9,10].

In order to improve the sensitivity and selectivity of biosensors, a variety of conductive materials or conductive nanomaterials have been added to the biosensing system, which mainly contain metal nanoparticles, metal oxide nanoparticles, carbon materials and conductive polymers. Metal nanoparticles have been widely applied in both chemical sensors and biosensors due to their high surface area, high mechanical strength, ultra-light weight, rich electronic properties, and excellent chemical and thermal stability [11]. However, the aggregation of metal nanoparticles extremely restrains their electrocatalytic properties. Combination of metal nanoparticles with nanofibers can effectively solve the aggregation problem. Bacteria cellulose (BC), as a green, economical, and low-cost biopolymer, is produced by *Acetobacter xylinum*, which usually forms netlike pellicles consisting of ultrafine nanofibers with widths less than 100 nm. BC possesses numerous unique properties, like high mechanical strength, high crystallinity, water absorbance, biocompatibility, polyfunctionality, and hydrophilicity [12]. Accordingly, metal nanoparticles-BC composites are suitable materials that can be applied in biosensors.

In this work, we synthesized Pd nanoparticles-BC (PdBC) hybrid nanofibers by *in-situ* chemical reduction method. The as-prepared PdBC nanofibers were further employed to modify electrode with Lac and Nafion to construct novel biosensing platform. The obtained biosensor showed excellent bioeletrocatalysis towards dopamine with high sensitivity, low detection limit and wide linear range. Moreover, the sensor demonstrated its practical application potential by detecting dopamine existing in human urine with satisfactory recovery. Our study offers the experimental and technological supports for exploiting high-efficient enzyme based biosensors for dopamine detection.

## 2. Results and Discussions

### 2.1. Characterization of PdBC Hybrid Nanofibers

**Figure 1:**
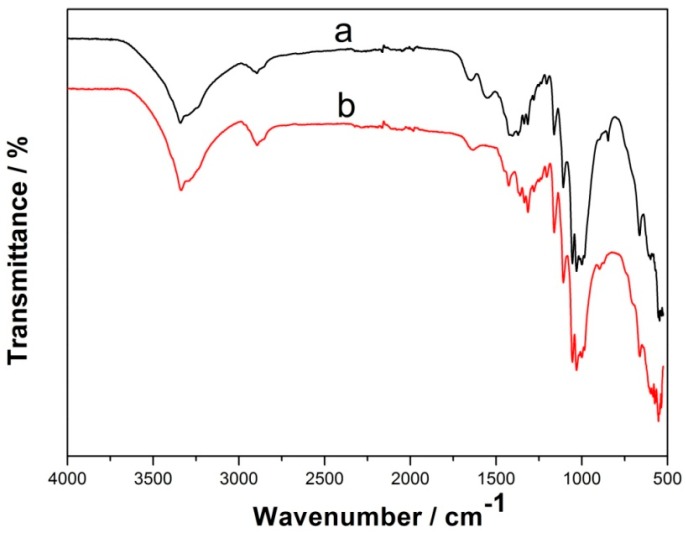
Fourier transform infrared (FT-IR) spectra of: BC (**a**); and PdBC (**b**).

Fourier transform infrared (FT-IR) spectra was employed to investigate the surface functional groups of BC and PdBC, and the result is shown in Figure 1. It is clear the BC and PdBC FT-IR spectra are almost similar. For the spectrum of BC (curve a), the characteristic band at about 3342 cm^−1^ was related to the stretching vibration of O-H, and the absorption peak at around 2895 cm^−1^ was ascribed to the stretching vibration of –CH_2_ [13]. In addition, the peaks at 1162 cm^−1^, 1107 cm^−1^, and 1055 cm^−1^ were due to the C-C stretching vibration, skeletal/vibrations and ring vibrations, respectively [14]. For the spectrum of PdBC (curve b), the locations of the characteristic bands only slightly shifted, indicating that the synthetic process of PdBC did not affect the functional groups of BC. The FT-IR analysis suggested that the PdBC nanofibers well maintained the chemical properties of BC.

**Figure 2:**
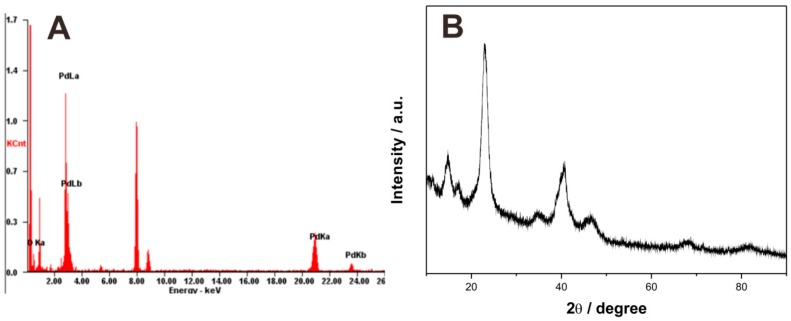
Energy dispersive X-ray (EDS) spectra (**A**); and X-ray diffraction (XRD) pattern (**B**) of PdBC.

Energy dispersive X-ray (EDX) spectroscopy characterization was used to determine the Pd element existing in the PdBC nanofibers. As shown in Figure 2A, there are several absorption peaks of Pd in the EDS spectra of PdBC, implying the existence of Pd element. X-ray diffraction (XRD) test was conducted to further study the chemical components of PdBC, and the result is displayed in Figure 2B. The diffraction peaks appearing at *ca.* 40.2°, 46.6°, and 68.2° corresponded to the (111), (200), and (220) plane reflections of palladium, respectively [15]. Besides, the peaks at *ca.* 14.8°, 17.2°, and 22.9° were related to the (1–10), (110) and (200) crystallographic planes of bacterial cellulose, respectively [16]. According to the Scherrer equation [17]:

(1) $$D=\frac{K\gamma }{B\cos\theta }$$

where *D* is the crystallite size, *K* is Scherrer constant, γ is the wavelength of X-ray radiation, *B* is the full width at half maximum (FWHM) of peak and θ corresponds to the peak position (in current study, 2θ = 40.2° for Pd). The average diameter of Pd nanoparticles was calculated to be *ca.* 4.7 nm. EDS and XRD characterizations jointly demonstrated the successful synthesis of palladium nanoparticles in the PdBC nanofibers.

**Figure 3:**
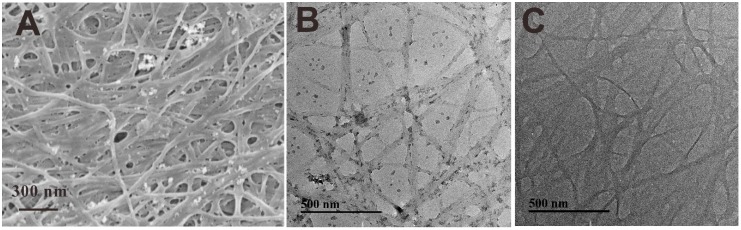
SEM image (**A**) of PdBC. TEM images of: PdBC (**B**); and BC (**C**).

The morphologies of PdBC and BC were observed by SEM and TEM. As can be seen in Figure 3A, the BC nanofibers formed a three-dimensional net-like structure with numerous palladium nanoparticles dispersing in the fibrous web. Some nanoparticles were attached on the fiber surfaces, while some others were aggregated together to become larger size of particles, which may be caused by the inter-attraction of the nanoparticles during the reduction reaction process. Figure 3B shows the TEM image of PdBC, where the tiny nanoparticles were loaded on the fiber surface. Meanwhile, some large nanoparticles were dropped from the BC nanofibers, which were caused by the ultrasonic dispersion in the preparation process of TEM sample. The average fiber diameter of BC nanofibers was about 35.6 nm, and the average diameter of Pd nanoparticles was around 13.8 nm, which was larger than the theoretical value calculated by Scherrer equation, indicating the aggregation of Pd nanoparticles. For comparison, the TEM image of BC is shown in Figure 3C, the BC nanofibers were randomly distributed with no impurities on them, suggesting their high purity.

**Figure 4:**
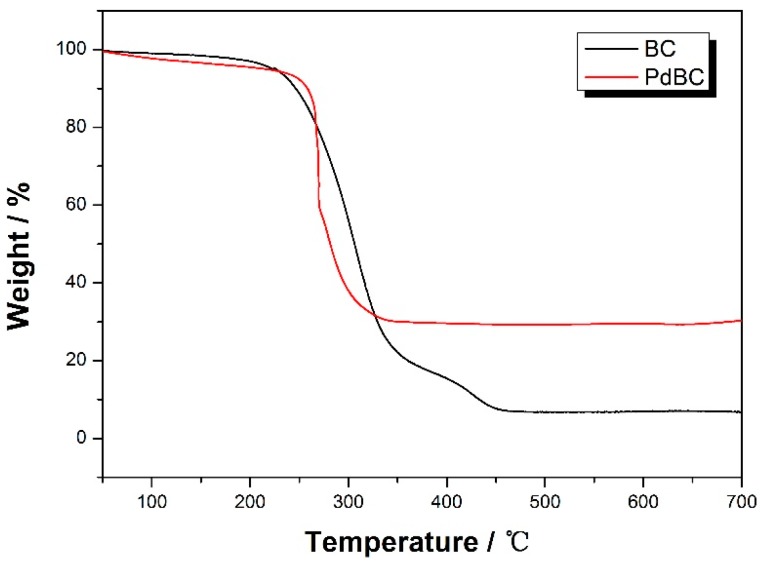
Thermogravimetric analysis (TGA) curve of BC nanofibers and PdBC nanofibers.

Figure 4 displays the thermogravimetric analysis (TGA) curve of BC nanofibers and PdBC nanofibers. For the PdBC nanofbiers, the sample showed two distinct weight losses. The first one occurred in the temperature range from 30 °C to 150 °C, which could be ascribed to the evaporation of water in PdBC nanofibers. The second one happened at around 240 °C, corresponding to the degradation of the main cellulose skeleton [18]. The weight loss of the PdBC nanofibers terminated at around 340 °C, the remaining products may be palladium oxide in consideration of oxidation of palladium nanoparticles. The content of palladium nanoparticles in the PdBC nanofibers could be calculated to be 28.8%. The BC nanofibers showed similar curve, while the weight of remaining products was only 6.8%, indicating the almost complete calcination of BC nanofibers.

### 2.2. Electrochemical Performance of Modified Electrodes

**Figure 5:**
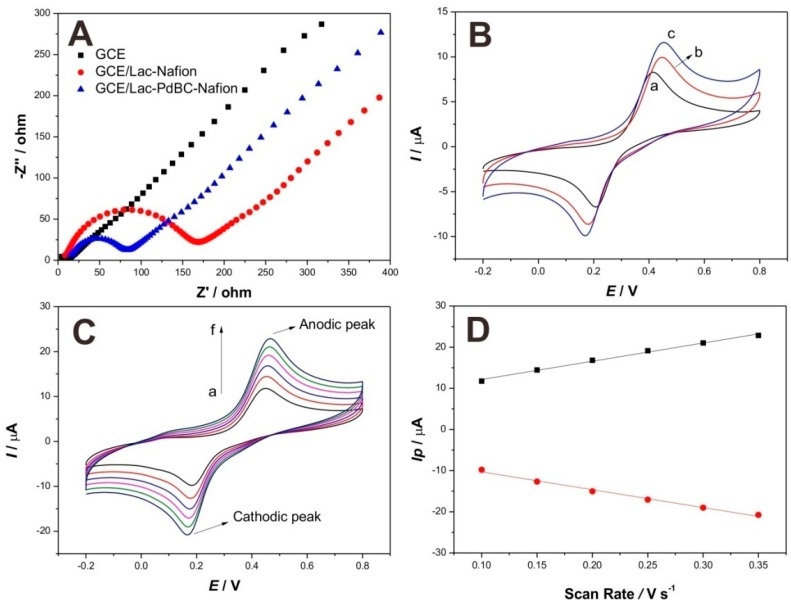
Electrochemical Impedance Spectroscopy (EIS) of modified electrodes: bare GCE, GCE/Lac-Nafion, and GCE/Lac-PdBC-Nafion in 0.1 M KCl containing 5 mM Fe(CN)_6_^3−/4−^. Frequency range: 0.01 Hz–100,000 Hz. Amplitude: 5 mV (**A**); Cyclic voltammograms of: (a) bare GCE; (b) GCE/Lac-Nafion; and (c) GCE/Lac-PdBC-Nafion in pH 7.0 PBS containing 100 µM dopamine at 100 mV·s^−1^ (**B**);Cyclic voltammograms of GCE/Lac-PdBC-Nafion in pH 7.0 PBS containing 100 µM dopamine at scan rates of 100, 150, 200, 250, 300, and 350 mV·s^−1^ (a–f), respectively (**C**); Plots of the corresponding anodic and cathodic peak currents *vs.* scan rate (**D**).

Electrochemical Impedance Spectroscopy (EIS) characterization was utilized to compare the interface resistances of different modified electrodes, the result of Nyquist polt of impedance is shown in Figure 5A. The values of charge transfer resistance (R_ct_) of modified electrodes are proportional to the diameters of semicircles. Apparently, bare electrode (GCE) shows almost a straight line, indicating the negligible R_ct_ value. However, an obvious semicircle occurred on the curve for GCE/Lac-Nafion, the R_ct_ value is about 168 Ω, suggesting that the interface resistance was increased by the immobilization process of Lac, while the semicircle diameter of the curve for GCE/Lac-PdBC-Nafion was smaller than that of GCE/Lac-Nafion, and the R_ct_ value also decreased to 78 Ω. This demonstrated that the PdBC nanofibers accelerated the electron transfer, weakened the interface resistance of modified electrode, which could be attributed to the good electron conductivity ability of palladium nanoparticles.

**Scheme 1:**
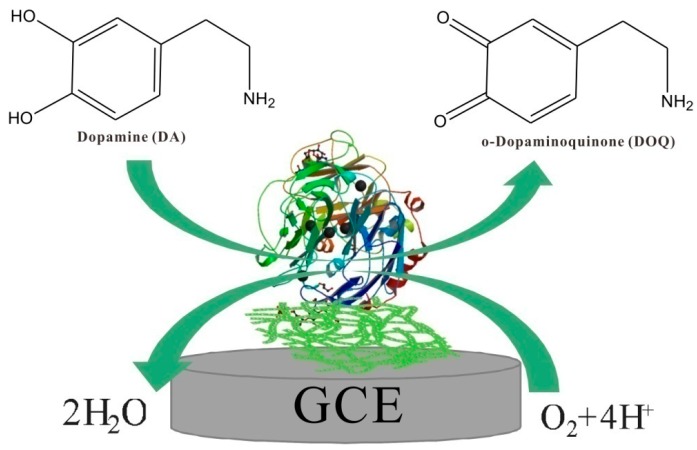
Schematic illustration of the catalyzed oxidation of dopamine by laccase on the electrode surface.

The electrochemical responses of three modified electrodes, including bare GCE, GCE/Lac-Nafion, and GCE/Lac-PdBC-Nafion, were compared by comparing the cyclic voltammograms of these electrodes in pH 7.0 PBS containing 100 µM dopamine. The result is shown in Figure 5B. These electrodes all show a pair of distinct redox peaks, which can be ascribed to the redox electrochemical reaction of dopamine happening on the electrode surface. It is observed that bare GCE possessed the smallest oxidation and reduction peak current values, indicating the poor electrochemical response of bare GCE. GCE/Lac-Nafion presented higher peak current values as compared to bare GCE, the oxidation peak current value reached to 10.03 µA, and the reduction peak current value was *ca.* 8.67 µA. This can be attributed to the highly efficient catalysis of Lac toward dopamine. It is noticeable that GCE/Lac-PdBC-Nafion shows the largest redox peak current values, which were increased to 11.59 µA and 9.88 µA, respectively. This demonstrated that the PdBC nanofibers can enhance the electrochemical response of modified electrode, maybe attributing to the good conductivity and the synergetic catalysis of palladium nanoparticles. The whole electrochemical reaction was a quasi-reversible cyclic process and the sensing mechanism of Lac on GCE/Lac-PdBC-Nafion is illustrated in Scheme 1. Under the presence of molecular oxygen, the dopamine (DA) was oxidized to o-Dopaminoquinone (DOQ) by Lac, coupled with the electrocatalytic reduction of oxygen to water on the surface of GCE. The reaction process can be described as follows:

DA + Lac(oxy) → DOQ + Lac(deoxy) + 2H^+^ + 2e^−^ (2)

Lac(deoxy) + O_2_ + 4H^+^ → Lac(oxy) + 2H_2_O (3)

Figure 5C shows the influence of scan rates on the cyclic voltammograms of GCE/Lac-PdBC-Nafion. As the scan rates grew from 100 to 350 mV·s^−1^, both of anodic peak and cathodic peak current values increased. As can be seen in Figure 5D, the peak current values enhanced linearly with the scan rates and were proportional to the scan rates. This indicated the electrochemical conduct occurring on the electrode surface was a surface-controlled electrochemical reaction process and the composite film was well attached on the electrode surface, which can be attributed to the excellent immobilization effect of Nafion.

### 2.3. Analytical Performance for Detecting Dopamine

**Figure 6:**
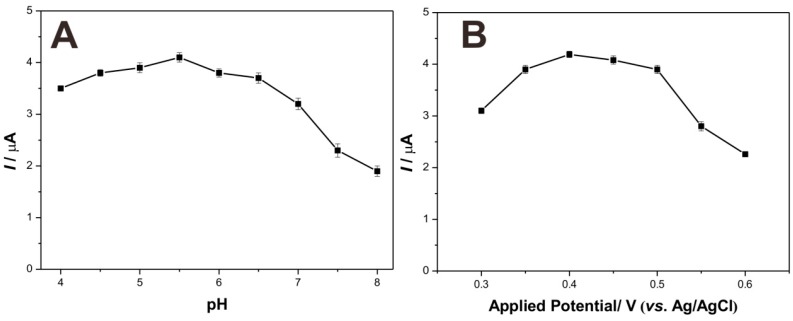
The effects of pH (**A**) and applied potentials (**B**) of GCE/Lac-PdBC-Nafion on the catalytic currents of dopamine in 0.1 M PBS containing 100 µM dopamine.

Chronoamperometry was employed to investigate the analytical performance of as-prepared biosensor for detecting dopamine. Before the amperometric tests, some parameters like solution pH and applied work voltage were optimized. As shown in Figure 6, the optimal pH and applied potential were pH 5.5 and 0.4 V, respectively. Considering the clinical application of biosensor, pH 7.0 and applied potential 0.4 V were fixed in the following chronoamperometry tests.

**Figure 7:**
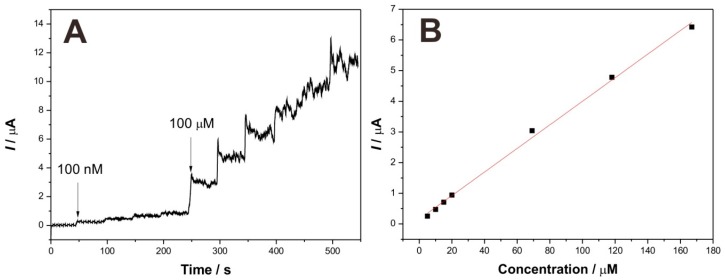
Typical current–time response curve of the GCE/Lac-PdBC-Nafion upon successive additions of dopamine into pH 7.0 PBS, applied potential: 0.4 V (**A**); Calibration curve of steady-state currents *vs.* dopamine concentrations (**B**).

Figure 7A depicts the typical current–time response curve of the GCE/Lac-PdBC-Nafion upon successive additions of dopamine into pH 7.0 PBS with 0.4 V of applied potential. Herein, two concentrations of dopamine solution (2 mM and 20 mM) were successively added into the acetate buffer solution. It can be clearly seen that once the 100 nM of dopamine was added, the response current increased instantly, the time for the current value reaching to the 95% of next maximum response current value was only 5 s, indicating a fast response of biosensor, which may be attributed to the easy diffusion of dopamine in the Lac-PdBC-Nafion composite film. Figure 7B shows the calibration curve of response currents *versus* dopamine concentrations. The current values increased linearly with the ascent of dopamine concentration. The linear range was from 5 µM to 167 µM with a correlation coefficient (*R*^2^) of 0.997 (*n* = 7). The sensitivity was 38.4 µA·mM^−1^ and the detection limit was estimated to be 1.26 µM at a signal-to-noise of 3 (S/N = 3). Table 1 compares the biosensing performance of dopamine sensors. Our biosensor showed satisfactory detection results toward dopamine with low detection limit, high sensitivity and wide linear range.

**Figure 8:**
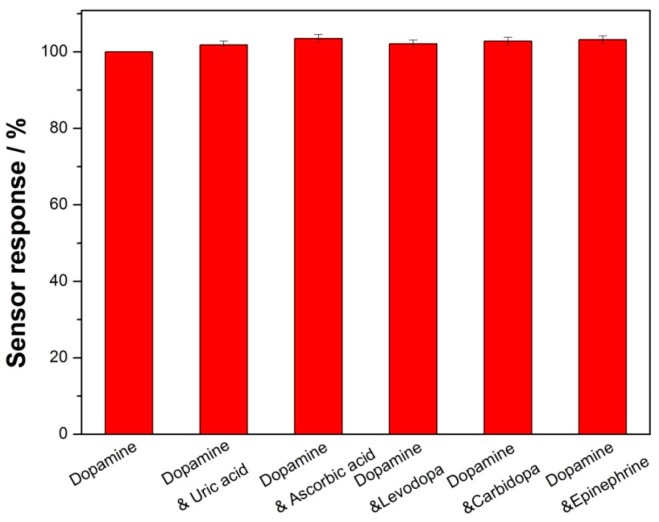
Selectivity of the GCE/Lac-PdBC-Nafion in pH 7.0 PBS containing different interferents.

**Figure 9:**
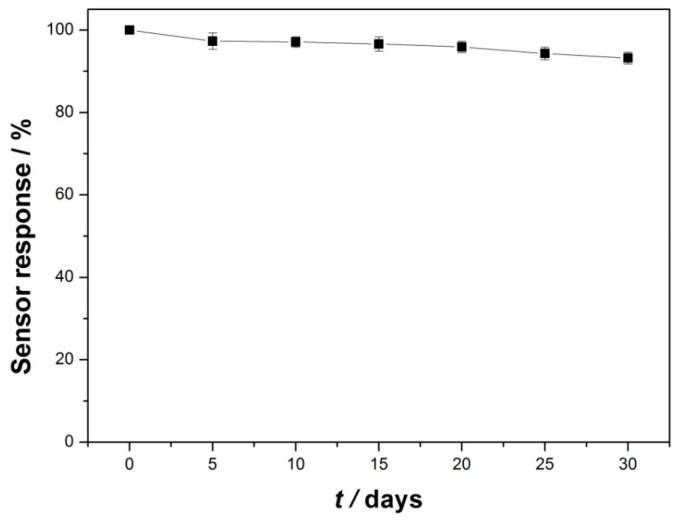
Storage stability of the GCE/Lac-PdBC-Nafion in pH 7.0 PBS at 4 °C.

The fabrication reproducibility of the GCE/Lac-PdBC-Nafion was investigated by successive detection of 100 µM dopamine by six modified electrodes prepared in the same way. The relative standard deviation (*RSD*) was 3.7%, implying the acceptable reproducibility of the GCE/Lac-PdBC-Nafion. The *RSD* of the GCE/Lac-PdBC-Nafion for 15 times of successive detection of 100 µM dopamine was 2.3%, indicating excellent repeatability of the GCE/Lac-PdBC-Nafion. The selectivity experimental result is shown in Figure 8. The current response of the GCE/Lac-PdBC-Nafion for 100 µM dopamine solution and 100 µM dopamine solution containing 100 µM interferents (uric acid, ascorbic acid, levodopa, carbidopa and epinephrine) was measured, respectively. Apparently, these interferents almost produced no effects on the current response of biosensor, indicating the good selectivity of the GCE/Lac-PdBC-Nafion. It can be seen from the Figure 9, the storage stability of the GCE/Lac-PdBC-Nafion in pH 7.0 PBS at 4 °C was satisfactory, through one month of storage, the response current value could retain 93.2% of original value, suggesting the great storage stability of the GCE/Lac-PdBC-Nafion.

### 2.4. Real Sample Analysis

To test the practical application of the biosensor, we conducted recovery experiments using human urine, which was 10-fold diluted with pH 7.0 PBS before analysis experiments. Then, 100 µM of dopamine was added into the as-prepared samples, which was named by *C*_added_. The determination content was then denominated *C*_found_. The result is shown in Table 2. It is seen that the recovery was very close to 100%, and the *RSD* was only 3.07%. This real sample analysis test demonstrated that the as-prepared biosensor can be successfully applied in the trace detection of dopamine in human urine.

## 3. Materials and Methods

### 3.1. Chemicals and Reagents

Laccase (Lac, enzyme activity ≥10 U/mg) from *Trametes versicolor* was purchased from Sigma-Aldrich (St Louis, MO, USA). Nafion (5% *w*/*w*) was obtained from Shanghai Branch, Du Pont China Holding Co., Ltd. (Shanghai, China). Palladium chloride (PdCl_2_) and dopamine were purchased from Shanghai Aladdin Chemical Reagent Company (Shanghai, China). All of the chemicals were analytical grade and used without further purification. In addition, 0.1 M phosphate buffer solution (PBS, pH = 7.0) was used as a supporting electrolyte. All aqueous solutions were prepared with deionized water (DIW).

### 3.2. Apparatus

Fourier transform infrared (FT-IR) spectra were recorded in the range of 500–4000 cm^−1^ on a Nicolet iS10 FT-IR spectrometer (Thermo Fisher Scientific, Waltham, MA, USA). The chemical components of PdBC nanofibers were analyzed by an Energy dispersive X-ray spectroscopy (EDX, EDAX-TSL, AMETEK, Hillsboro, OR, USA) and a Powder D8 Advance X-ray diffraction (XRD, Bruker AXS D8Coventry, UK). Thermogravimetric analysis (TGA) measurement was conducted using a Mettler Toledo analyzer (Mettler Toledo, Shanghai, China) in air atmosphere, the temperature range was from ambient temperature to 700 °C with a heating rate of 10 °C/min. The morphologies of PdBC nanofibers were observed using a scanning electron microscope (SEM, Hitachi SU1510, Tokyo, Japan) and a high-resolution transmission electron microscope (TEM, JEOL/JEM-2100, Tokyo, Japan). Prior to scanning under the SEM, the samples were sputter coated with gold for 90 s to avoid charge accumulations. Nano Measurer software (Malvern, Manchester, UK) was used to measure the diameters of the BC nanofibers and Pd nanoparticles. Electrochemical experiments were conducted at room temperature using a CHI 660E electrochemical workstation (CH Instruments, Inc., Shanghai, China). A three-electrode cell with a glass carbon electrode (GCE) (3.0 mm in diameter, purchased from Gaoss Union Technology Co., Ltd., Wuhan, China), a platinum wire auxiliary electrode and an Ag/AgCl reference electrode was used for electrochemical measurements. The electrolyte solution was bubbled with highly pure nitrogen for 15 min before electrochemical experiments and a nitrogen atmosphere was kept for the solution throughout the experiments except the amperometric experiments.

### 3.3. Synthesis of PdBC Hybrid Nanofibers

*Gluconacetobacter xylinum* was kindly provided by Donghua Univerisity (Shanghai, China) and used to produce the bacterial cellulose pellicles. The bacterium was cultured on Hestrin and Schramm (HS) medium by staticing incubation. The medium was composed of 5% (*w*/*v*) glucose, 1.6% (*w*/*v*) bacto-peptone, 0.2% (*w*/*v*) citric acid, 0.2% (*w*/*v*) disodium hydrogen phosphate, 0.3% (*w*/*v*) potassium dihydrogen phosphate, and 0.03% (*w*/*v*) magnesium sulfate. These culture media were sterilized at 120 °C in autoclave for 2 h by autoclaving. Cells pre-cultured in a test tube containing a small cellulose pellicle on the surface of the medium were inoculated into a 100 mL Erlenmeyer flask containing 10 mL of the HS medium. The flask was incubated statically at 30 °C for 7 days. The synthesized cellulose were dipped into 1% sodium hydroxide solution for 2 h at 80 °C in order to eliminate the cells and components of the culture liquid, and then rinsed 3 times to pH 7 in deionized water.

The PdBC hybrid nanofibers were synthesized by *in-situ* chemical reduction method. The as-prepared bacterial cellulose (BC) was disrupted by Ultrasonic Cell Disruption System for 10 min to disperse the BC. Then the BC was immersed in an aqueous mixture solution containing saturated PdCl_2_ metal salt at 40 °C for 24 h. After that, 0.01 M KBH_4_ solution was added to reduce the palladium salt to obtain Pd nanoparticles. The final products were rinsed and dried.

### 3.4. Preparation of Biosensors

Considering the optimal response and stability of modified electrode, among control-experiments, the concentration and mass ratio of Nafion, PdBC nanofibers and Lac were optimized. The preparation procedure of biosensors are described as follows: 1.5 mg of PdBC nanofibers were added into 5 mL of pH 7.0 PBS with the aid of ultrasonication and stirring to obtain PdBC suspension. Subsequently, 15 mg of Lac and 75 µL of Nafion solution (5 wt %) were added into the above PdBC suspension, by which the final mixture suspension was achieved. Eventually, 10 µL of the mixture was dropped on a freshly polished glass carbon electrode (GCE) surface to fabricate the biosensor, and the dried modified GCE was named GCE/Lac-PdBC-Nafion, which was stored at 4 °C for use.

GCE/Lac-Nafion modified electrode was fabricated by similar methods with same amount of Lac for comparison experiments. All the modified electrodes were immersed into buffer for 30 min to remove impurities before electrochemical measurements.

## 4. Conclusions

In summary, PdBC hybrid nanofibers were synthesized by *in-situ* chemical reduction method. The Pd nanoparticles were observed to be evenly dispersed on the surfaces of BC nanofibers, which is beneficial for developing their electrocatalytic effect. A sensitive biosensor was fabricated by modifying a mixture containing PdBC nanofibers, Lac, and Nafion on electrode surface. The as-prepared biosensor showed great biological electrocatalysis towards dopamine with high sensitivity, low detection limit and wide linear range. Besides, the sensor was successfully used in dopamine detection in human urine. This novel electrochemical biosensor provided a promising method for dopamine analysis in clinical application.

## Figures and Tables

**Table 1:** Sensing performance comparison of different sensors toward dopamine.

Electrodes	Detection Limit (µM)	Linear Range (µM)	Sensitivity (µA·mM^−1^)	Ref.
SiO_2_/C/Cu(II)phtalocyanine	0.6	10–140	0.63	[19]
SPE/MWCNT/β-CD/GOx	3.14	5–35	4.4	[20]
GNS/PEI/AuNP/GCE	0.2	2–48	264	[21]
RGO-Pd-NPs/GCE	0.233	1–150	2.62	[22]
Lac/SiO_2_-PA/GCE	0.26	0.99–103.1	1.57	[23]
GCE/Lac-PdBC-Nafion	1.26	5–167	38.4	This work

**Table 2:** Determination of dopamine content in human urine (*n* = 5).

Sample	*C*_added_ (µM)	*C*_found_ (µM)	Recovery (%)	RSD (%)
Human urine	100.00	105.13	105.13	3.07
		97.89	97.89	
		101.27	101.27	
		98.34	98.34	
		103.23	103.23	
